# Radical zinc-atom-transfer-based carbozincation of haloalkynes with dialkylzincs

**DOI:** 10.3762/bjoc.9.28

**Published:** 2013-02-04

**Authors:** Fabrice Chemla, Florian Dulong, Franck Ferreira, Alejandro Pérez-Luna

**Affiliations:** 1 Institut Parisien de Chimie Moléculaire (UMR 7201), FR 2769, UPMC Univ Paris 06, CNRS, Bâtiment F 2ème et., Case 183, 4 place Jussieu, F-75005 Paris, France

**Keywords:** carbenoids, carbometallation, carbozincation, radicals, tandem reaction

## Abstract

The formation of alkylidenezinc carbenoids by 1,4-addition/carbozincation of dialkylzincs or alkyl iodides based on zinc atom radical transfer, in the presence of dimethylzinc with β-(propargyloxy)enoates having pendant iodo- and bromoalkynes, is disclosed. Formation of the carbenoid intermediate is fully stereoselective at −30 °C and arises from a formal *anti*-selective carbozincation reaction. Upon warming, the zinc carbenoid is stereochemically labile and isomerizes to its more stable form.

## Introduction

The last few years have witnessed a gaining interest in the use of organozinc reagents as nontoxic radical precursors or mediators [1–3]. As part of this development, the so-called radical-polar reactions in which alkylzinc reagents are used as mediators in a radical transformation that affords a new zincated species, have emerged as valuable tools in synthesis. Pivotal to the processes disclosed so far using alkylzinc derivatives is zinc atom radical transfer [4]. In general terms, the reaction involves a radical chain process initiated by the formation of an alkyl radical from the organozinc derivative in the presence of oxygen [5–14]. The newly formed radical then undergoes one or more radical transformations before being reduced by the alkylzinc reagent through homolytic substitution at zinc, producing a new organozinc derivative along with an alkyl radical that sustains a radical chain. Overall, the in situ transformation of simple organozinc reagents into more elaborate ones is thus achieved, and subsequent reaction with electrophiles is possible [15–30].

More specifically, building on well-established addition reactions of carbon-centered radicals to carbon–carbon double and triple bonds, such reactivity has been advantageously employed in the context of carbozincation chemistry [31]. The intramolecular carbozincation of unactivated terminal alkenes following zinc atom transfer processes, including a 5-*exo*-*trig* cyclization step, has been reported. This is, for instance, the case in the formation of (pyrrolidylmethyl)zinc and (tetrahydrofuranylmethyl)zinc derivatives by reaction of dialkylzinc, organozinc and copper–zinc mixed reagents with (*N*-allyl)aminoenoates [32–34] and β-(allyloxy)enoates [35], in the formation of (pyrrolidonylmethyl)zinc by condensation of dialkylzincs with *N*,*N*-diallylpropiolamide [36], and also in the cyclization of alkenylzinc iodides to cyclopentylmethylzinc iodides, formerly believed to be anionic in nature [4]. Carbozincations of alkynes based on zinc atom transfer have also been disclosed. The reaction of dialkylzincs or of alkyl iodides in the presence of Me_2_Zn/O_2_ with β-(propargyloxy)enoates entails the intramolecular carbometallation of the pendant alkynes substituted by silyl, alkyl, aryl, alkenyl or amino groups by a 5-exo *dig* radical cyclization step [37–38]. Intermolecular carbozincation of terminal arylacetylenes [39] and of diethyl acetylenedicarboxylate [40] has been achieved by dialkylzinc-mediated radical additions. Worthy of note is that in some cases the zinc-atom-transfer-based carbozincation of alkynes can occur with *anti* selectivity [38,40], and thereby represents a complementary approach to transition-metal-mediated carbozincations, which are generally *syn*-selective [41–46].

To explore further the possibilities offered by zinc atom transfer processes we considered the possibility to prepare alkylidenezinc carbenoids by radical-based carbozincation of haloalkynes. Such carbenoids are multipurpose reagents [47] that are typically prepared from 1,1'-dihaloalkenes, either by lithium/halogen exchange followed by transmetallation with zinc salts or by direct zinc/halogen exchange [48–50]. Alternatively, they can also be prepared efficiently by selective monohalogenation of alkylidene *gem*-bismetallic intermediates [51].

**Scheme 1:**
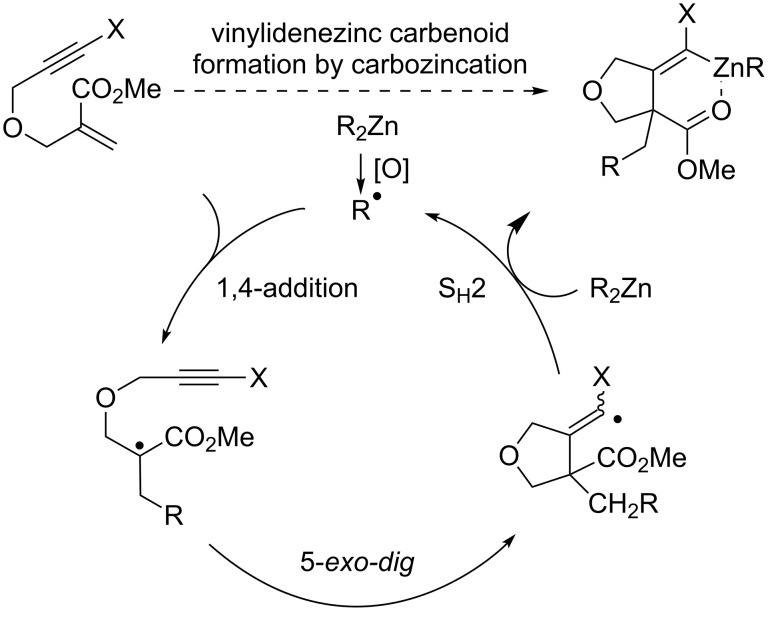
Anticipated formation of alkylidene zinc carbenoids by reaction of dialkylzincs with β-(propargyloxy)enoates having pendant haloalkynes.

To the best of our knowledge, the preparation of alkylidenezinc carbenoids by the direct carbozincation of haloalkynes has not been reported [52]. As a starting point to develop such an approach, we reasoned that the reaction of dialkylzincs with β-(propargyloxy)enoates bearing pendant haloalkynes would be ideally suited (Scheme 1). On the one hand it would provide a means to control totally the regioselectivity of the radical addition to the haloalkyne, and on the other hand the envisioned zinc atom transfer to an α-halo vinylic radical should be favorable as a result of the presence of the ester moiety. Hereafter, we disclose our findings concerning this reaction.

## Results and Discussion

**Scheme 2:**
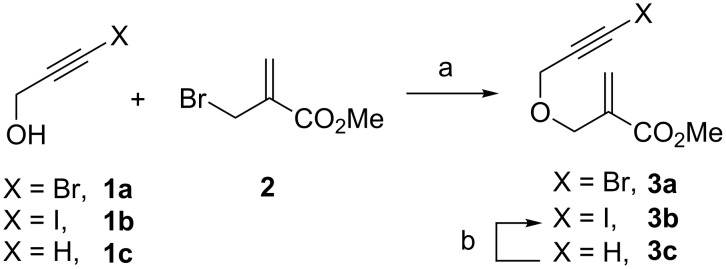
Preparation of β-(propargyloxy)enoates having pendant haloalkynes. Reagents and conditions: (a) **2** (1.4 equiv), Et_3_N (4 equiv), NaI (10 mol %), CH_2_Cl_2_, 50 °C, sealed tube, 74% (**3a**), 87% (**3c**); (b) AgNO_3_ (1.5 equiv), *N*-iodosuccinimide (1.5 equiv), acetone, rt, 53% (**3b**).

β-(Propargyloxy)enoates **3a** and **3b** having a pendant bromoalkyne and an iodoalkyne moiety, respectively, were prepared by condensation of propargylic alcohols **1** with methyl 2-(bromomethyl)acrylate (**2**) (Scheme 2). Enoate **3a** was readily obtained by direct reaction of 3-bromopropargyl alcohol (**1a**). By contrast, the reaction of the iodo analogue **1b** with **2** proved troublesome as it led to inseparable mixtures of the desired enoate **3b** and non-iodinated enoate **3c**. Thus, **3b** was best prepared by iodinating (AgNO_3_/NIS) the terminal alkyne of enoate **3c** prepared from propargyl alcohol (**1c**) and acrylate **2**.

According to our previously optimized conditions for the 1,4-addition/carbozincation reaction of dialkylzincs with β-(propargyloxy)enoates [37–38], bromoalkyne **3a** was treated with Et_2_Zn at room temperature in Et_2_O under an argon atmosphere (Table 1, entry 1). To our delight, following acidic work-up, the expected methylenetetrahydrofuranyl bromide **4aa** was obtained in 43% isolated yield as a mixture of diastereoisomers in a 77:23 *Z*/*E* ratio [53]. Hydrolysis with D_2_O evidenced the intermediate formation of an alkylidene zinc carbenoid as deuterated **4aa**-**D** was produced (Table 1, entry 2). As previously noted in the case of similar 1,4-addition/carbozincation sequences [37–38], deuterium incorporation was nearly quantitative for the *Z* isomer, and much lower for the *E* one. More unexpectedly, however, 40% of alkylidenetetrahydrofuran **5a**, wherein the bromine atom had been substituted by an ethyl group, was also isolated as a 79:21 *Z*/*E* mixture. Deuterium labeled **5a-D** was produced following hydrolysis with D_2_O (Table 1, entry 2), thereby showing that an alkylidenezinc intermediate was being formed in the generation of this side-product under these reaction conditions.

**Table 1:** 1,4-addition/carbozincation of dialkylzincs with β-(propargyloxy)enoates **3** having pendant haloalkynes in the presence of traces of air.^a^

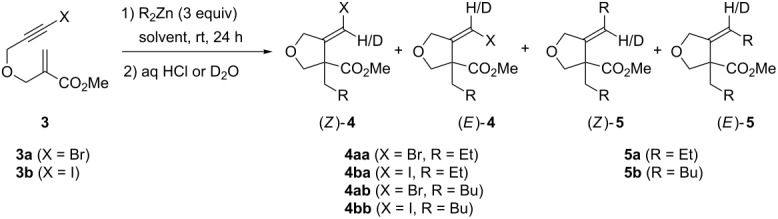					

Entry	Enoate	X	Solvent	R	Products (Yield^b^ (%) [dr^c^ (*Z*/*E*)])

1	**3a**	Br	Et_2_O	Et	**4aa** (43 [77:23]); **5a** (40 [79:21])
2	**3a**	Br	Et_2_O	Et	**4aa-D**^d^ (44 [81(90% D):19(10% D)]); **5a-D**^d^ (35 [84(85% D):16(40% D)])
3	**3b**	I	Et_2_O	Et	**4ba** (40^e^ [98:2]); **5a** (21^e^ [82:18])
4	**3a**	Br	Et_2_O	*n*-Bu	**4ab** (76 [70:30]); **5b** (7 [nd^f^])
5	**3b**	I	Et_2_O	*n*-Bu	**4bb** (58 [98:2])
6	**3a**	Br	CH_2_Cl_2_	Et	**4aa** (39^d^ [78:22]); **5a** (17^d^ [91:9])
7	**3a**	Br	CH_2_Cl_2_	*n*-Bu	**4ab** (37 [76:24]); **5b** (12 [nd]^f^)

^a^Reaction conditions : R_2_Zn (3 equiv), rt, 24 h under Ar atmosphere (see Experimental section). ^b^Combined yield of diastereomers after chromatography unless otherwise noted. ^c^Determined by ^1^H NMR analysis of the crude material. ^d^The reaction mixture was quenched with D_2_O. The percentage of deuterium incorporation is given in parenthesis for each compound. ^e^Determined by ^1^H NMR spectroscopy based on analysis of the crude mixture with biphenyl as internal standard. ^f^Not determined.

When iodoalkyne **3b** was used, a similar 40% yield of vinylic iodide **4ba** was obtained, but this time exclusively as the *Z* isomer (Table 1, entry 3). **5a** was also produced, but in a lower 21% yield and similar diastereoselectivity (82:18 *Z*/*E* ratio). Significantly lower levels of side-product formation arising from halogen substitution were observed when *n-*Bu_2_Zn was used, thus leading to improved results (Table 1, entries 4 and 5). The reaction with bromoalkyne **3a** provided vinylic bromide **4ab** in 76% yield and 70:30 *Z*/*E* ratio and only 7% of **5b**. Better, the reaction with iodoalkyne **3b** afforded exclusively iodide **4bb** in 58% yield and complete diastereoselectivity in favor of the (*Z*) isomer. Formation of substitution side-products was also diminished when CH_2_Cl_2_ was used as the solvent instead of Et_2_O, even though this had little impact on the efficiency and diastereoselectivity of vinyl halide formation (Table 1, entries 6 and 7). Reaction of Et_2_Zn with **3a** provided vinyl bromide **4aa** in 39% yield (78:22 *Z*/*E* ratio) and **5a** in 17% yield, while reaction of *n*-Bu_2_Zn gave **4ab** in 37% yield (76:24 *Z*/*E* ratio) and **5b** in 12% yield. It is worthy of note that no difference was observed between the different dialkylzincs in this case.

**Scheme 3:**
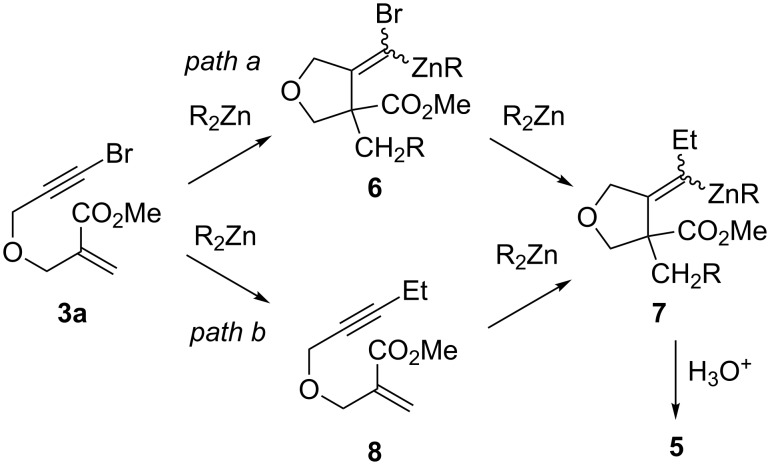
Possible reaction pathways to account for the formation of product **5**.

The formation of alkylidenezinc derivatives **7** leading to compounds **5** is intriguing (Scheme 3). A first possible mechanistic route could involve the reaction of zinc carbenoid **6** and the excess of dialkylzinc reagent via the intermediate formation of a zincate [48–51] (Scheme 3, path a). The stereoselectivity of such rearrangements is often dependent on the substrate structure, so the diastereopurity of **5** is not necessarily informative about that of **6** [48–51]. An alternative possibility to account for the formation of **7** could be the reaction of the dialkylzinc reagent with enoate **8** arising from a prior substitution of bromoalkyne **3a** with the dialkylzinc reagent (Scheme 3, path b). Both the diastereoselectivity and the levels of deuterium incorporation are very close to those obtained for the reaction of diethylzinc with pure **8** [37], which argues in favor of this mechanistic scenario.

**Scheme 4:**
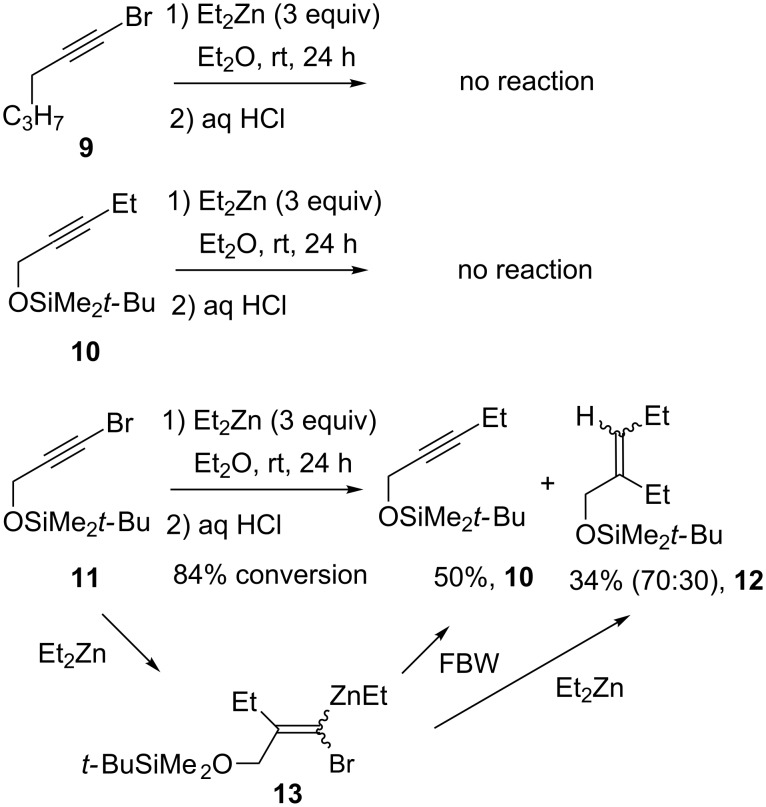
Test experiments to gain insight into the mechanism of formation of alkylidene zinc intermediate **7**.

To try to discriminate between these possibilities we conducted some additional test experiments (Scheme 4). In agreement with the general consideration that dialkylzinc reagents do not undergo uncatalyzed cross-coupling reactions with bromoalkynes, no reaction was observed between Et_2_Zn and 1-bromohexyne (**9**) [54]. By contrast, bromoalkyne **11** having a silyloxy group at the propargylic position reacted smoothly to afford ethyl-substituted alkyne **10** along with alkene **12**, which had incorporated two ethyl groups. **12** was isolated as a mixture of diastereoisomers in 70:30 dr. The fact that no reaction takes place between pure **10** and Et_2_Zn indicates that **12** is not formed by carbozincation. Hence, most likely it is formed by the reaction of Et_2_Zn and carbenoid **13** arising from the carbozincation of **11** (Scheme 4). Moreover, if **13** is indeed formed, it would also lead to alkyne **10** following Fritsch–Buttenberg–Wieschell (FBW) rearrangement [55–57]. Since the presence of the oxygen atom in the propargylic position should facilitate the carbometallation reaction [58], this mechanistic pathway provides a plausible explanation for the fact that bromine substitution occurs from α-oxgenated bromoalkyne **11** and not from **9**.

Regarding our 1,4 addition/carbocyclization sequence, these test experiments provide two important pieces of evidence for the behavior of **3a** in the presence of a dialkylzinc. First, β-alkoxy bromoalkynes undergo direct substitution with Et_2_Zn to some extent. Second, alkylidenezinc carbenoids react with dialkylzincs to afford the bromine substitution product. Thus, formation of alkylidenezinc compound **7** (and thereby **5**) most probably arises from both depicted mechanistic pathways (paths a and b, Scheme 3). In such a situation, we reasoned that in both possibilities, reducing the reaction time would limit the production of the unwanted side-products by limiting the contact time between the dialkylzinc reagent and either the starting bromoalkyne or the generated zinc carbenoid. Thus, we considered adding air to the reaction media in order to accelerate the oxidation of the dialkylzinc species and therefore radical production (Table 2).

**Table 2:** 1,4-Addition/carbozincation of dialkylzincs on **3a** in the presence of added air.^a^

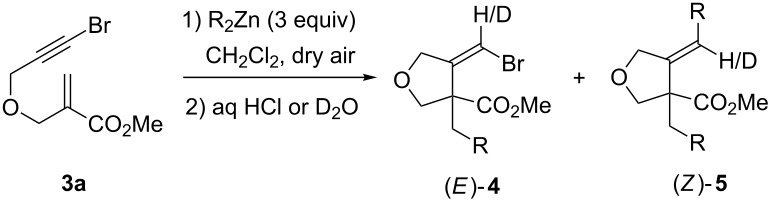					

Entry	R	Reaction conditions	Products [ratio]	Yield^b^ (%)	dr^c^ of product **4** (*E/Z*)

1	Et	rt, 1 h	**4aa**/(*Z*)-**5a** [84:16]	69^d^	54:46
2	Et	0 °C, 1 h	**4aa**	93	87:13
3	Et	−30 °C, 1 h	**4aa**	89	>98:2
4	Et	−30 °C, 1 h	**4aa-D**^e^	95^d^	>98(83% D):2
5	Et	−30 °C, 1 h, DCE^f^	**4aa-D**^e^	89	>98(83% D):2
6	Bu	−30 °C, 1 h	**4ab**	93	>98:2
7	Et	−30 °C, 1 h then rt, 24h	**4aa**/(*Z*)-**5a** [78:22]	78^d^	44:56
8	Et	−30 °C, 1 h then rt, 24 h	**4aa-D**^e^/(*Z*)-**5a-D**^g^ [78:22]	78^d^	44(<10% D):56(<10% D)

^a^Reaction conditions : R_2_Zn (3 equiv), CH_2_Cl_2_, dry air was bubbled at once into the reaction mixture, which was then kept under Ar atmosphere (see Experimental section). ^b^Combined yield of products after chromatography unless otherwise noted. ^c^Determined by ^1^H NMR analysis of the crude material. ^d^Determined by ^1^H NMR spectroscopy based on analysis of the crude mixture with biphenyl as the internal standard. ^e^The reaction mixture was quenched with D_2_O. The percentage of deuterium incorporation is given in parenthesis for each isomer. ^f^DCE = 1,2-dichloroethane was used as solvent instead of CH_2_Cl_2_. ^g^30% deuterium incorporation was observed for product **5a-D**.

A reduced amount of side-product **5** was indeed observed in the reaction of enoate **3a** with Et_2_Zn in CH_2_Cl_2_ at room temperature in the presence of added dry air (Table 2, entry 1). A mixture of vinyl bromide **4aa** and alkene **5a** in a 84:16 ratio and in 69% overall yield was obtained. However the diastereoselectivity of the formation of **4aa** dropped significantly. Lowering the reaction temperature had a highly beneficial impact on the reaction outcome. At 0 °C, the formation of **5a** was totally suppressed, and **4aa** was obtained with a much better diastereoselectivity, though remarkably in favor of the *E* isomer (Table 2, entry 2). At −30 °C, the exclusive and totally diastereoselective formation of (*E*)-**4aa** in excellent 89% isolated yield was obtained (Table 2, entry 3). Hydrolysis with D_2_O led to (*E*)-**4aa-D** with 83% deuterium incorporation when either CH_2_Cl_2_ or DCE were used as solvent (entries 4 and 5), therefore evidencing the intermediate stereoselective formation of an alkylidene zinc carbenoid. Similar results were obtained by using *n*-Bu_2_Zn, indicating that the process is quite general (Table 2, entry 6).

The diastereoselectivity of the formation of **4aa** seemed to be dependent not only on the reaction temperature, but also on the total reaction time (compare Table 1, entry 6 and Table 2, entry 1). Suspecting a possible *Z*/*E* isomerization of the alkylidenezinc carbenoid intermediate, we conducted an experiment wherein air was added to a mixture of enoate **3a** and Et_2_Zn in CH_2_Cl_2_ at −30 °C, and the reaction was first kept for 1 h at this temperature and then for 23 h at room temperature (Table 2, entry 7). Following acidic quench, very similar results to those noted for the same reaction carried out at room temperature (Table 2, entry 1) were observed. **4aa** was recovered in 61% yield as a 56:44 mixture of isomers along with alkene (*Z*)-**5a** in 22% yield. It is worthy to note that very low levels of deuterium incorporation were observed in this case.

These results have a three-fold consequence. First, they indicate that *Z*/*E* isomerization of the alkylidenezinc carbenoid occurs between −30 °C and room temperature. Second, it demonstrates that alkene **5a** can be formed by the reaction between the zinc carbenoid and Et_2_Zn (Scheme 3, path a), and that in such a case the transformation is stereoselective. Third, when oxygen is introduced into the reaction media, the alkylidenezinc carbenoid is eventually demetallated upon standing at room temperature.

**Scheme 5:**
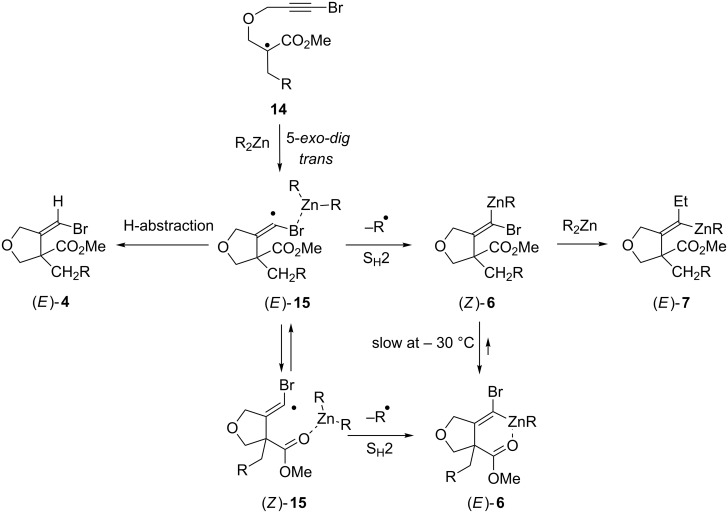
Mechanistic rationale for the reaction of dialkylzincs with β-(propargyloxy)enoate **3a**.

The different results obtained for the 1,4-addition/carbozincation of enoates having pendant bromoalkynes are consistent with our anticipated zinc atom radical transfer mechanism (Scheme 1) and can be rationalized according to the following scenario (Scheme 5). The process involves the initial 1,4-addition of radical R to the starting enoate and the subsequent 5-*exo-dig* cyclisation of enoxy radical **14** to provide α-bromovinyl radical **15** of *E* geometry. Substitution by electron-withdrawing groups slows down *E* to *Z* isomerization of vinylic radicals, and therefore, due to the presence of the bromine atom, interconversion of (*E*)-**15** into (*Z*)-**15** should not be fast. Thus, (*E*)-**15** reacts by Zn atom transfer prior to its equilibration to provide stereoselectively carbenoid (*Z*)-**6** [59] and to some extent by H-atom transfer to give reduced bromoalkene (*E*)-**4** [60]. Upon warming, (*Z*)-**6** isomerizes to its more stable isomer (*E*)-**6** wherein the zinc atom is coordinated intramolecularly to the adjacent ester. To a minor extent, reaction with the excess dialkylzinc present in the reaction media provides alkylidenezinc (*E*)-**7** stereoselectively. Note, however, that in the case where reactions are conducted at room temperature, *E*/*Z* equilibration of the intermediate radical **15** should be faster and zinc atom transfer from (*Z*)-**15** may also contribute to the formation of (*E*)-**6**.

Two situations are next to be distinguished. Under the conditions involving excess air, carbenoid **6** is protonated in the reaction media (even though we have not identified the proton source). It is possible that protonation occurs prior to full *E* to *Z* isomerization and vinyl bromide **4** is obtained in low diastereoselectivity. Under the conditions involving only a trace of air, after 24 h at room temperature, carbenoid **6** is still present and total *Z* to *E* isomerization has occurred. The reason why vinyl bromide **4** is only isolated with moderate diastereoselectivity following hydrolysis, is that (*E*)-**4**, formed from (*E*)-**15** by H-abstraction, is present from the start.

We finally considered the prospect to carry out the carbozincation of bromoalkynes using a combination of an alkyl iodide and dimethylzinc (Table 3). Towards this end, we first treated enoate **3a** with Me_2_Zn, following our previously reported optimized conditions for the 1,4-addition/carbozincation of β-(propargyloxy)enoates with Me_2_Zn [38], which proceeds in CH_2_Cl_2_ at 0 °C and under continuous introduction of dry air over a period of 1 h (Table 3, entry 1). After acidic quench, vinylic bromide (*E*)-**4ac** [61] was isolated in 77% yield as a single diastereoisomer. The stereoselective formation of an alkylidenezinc carbenoid intermediate was this time evidenced by treating the reaction mixture with iodine (Table 3, entry 2). Vinylic dihalide **16** was isolated in 64% yield, again as a single *Z* diastereoisomer. Thus, Me_2_Zn showed a similar reactivity to its higher homologues Et_2_Zn and *n*-Bu_2_Zn, with the additional advantage that no direct formation of vinyl bromide (*E*)-**4ac** by hydrogen abstraction was observed.

**Table 3:** Me_2_Zn-mediated 1,4-addition/carbozincation of alkyl iodides with **3a** in the presence of added air.^a^

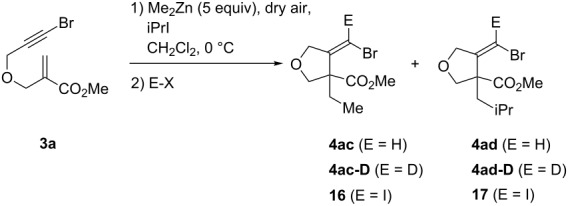				

Entry	iPrI (equiv)	E-X	Yield^b^ (%)	Products [ratio]

1	0	H_2_O	77	**4ac^c^**
2	0	I_2_	64	**16**
3^d^	5	H_2_O	59	**4ad**/**4ac** [87:13]
4^d^	10	H_2_O	87	**4ad**/**4ac**/**17** [75:6:19]
5^d^	10	D_2_O	75	**4ad-D**/**4ac-D**/**17** [75 (95% D):6 (95% D):19]

^a^Reaction conditions: Me_2_Zn (5 equiv), iPrI (equiv), CH_2_Cl_2_, 0 °C, 20 mL dry air was bubbled during 1 h into the reaction mixture via a syringe pump. ^b^Combined yield of isolated products after chromatography unless otherwise noted. ^c^The product was contaminated with ~10% of product resulting from the addition of the dichloromethyl radical (**4ae**, R = CHCl_2_). ^d^3 equiv Me_2_Zn were used.

In the presence of 5 equiv iPrI, a mixture of two (diastereomerically pure) compounds **4ad** and **4ac** in 87:13 ratio was observed. Incorporation of the iPr moiety was therefore the major reaction pathway. The competitive addition of a Me group could be reduced by increasing the amount of iPrI to 10 equiv (Table 3, entries 3 and 4). However, in this case, significant amounts of vinylic dihalide **17** were also isolated. Thus, if large amounts of iodide are used, radical iodine atom transfer between iPrI and the α-bromovinylic radical **15** resulting from the cyclization step (Scheme 5) becomes competitive with the desired zinc atom transfer and hampers the carbozincation process.

## Conclusion

In conclusion, we have shown that β-(propargyloxy)enoates having pendant iodo- and bromoalkynes undergo a 1,4-addition/carbozincation sequence by reaction with dialkylzincs or with alkyliodides in the presence of dimethylzinc. The sequence involves a radical chain mechanism initiated by air and provides the proof of concept that alkylidenezinc carbenoids can be prepared by carbozincation based on zinc atom transfer. In the disclosed process, we have demonstrated that the formation of a bromocarbenoid intermediate is fully stereoselective at −30 °C and arises from a formal *anti-*selective carbozincation reaction. Upon warming, the zinc carbenoid is stereochemically labile and isomerizes to its more stable form. In the absence of added air, no decomposition of the carbenoid intermediate is observed at room temperature for at least 24 h. Deuterium labeling and iodolysis experiments evidence that the zinc carbenoids prepared under such reactions can act as typical nucleophiles and should, thus, be well-suited for subsequent functionalization [47–51]. Furthermore, as indicated with the formation of some side-products observed during this work, they should also react readily as electrophiles toward organometallic nucleophiles and undergo intramolecular nucleophilic substitution reactions [47–51].

## Experimental

Experiments involving organometallic compounds were carried out in dried glassware under a positive pressure of dry Ar. All solvents were distilled to remove stabilizers and dried with a MBRAUN Solvent Purification System SPS-800. *n*-Bu_2_Zn (Fluka, ~1 N in heptane), Et_2_Zn (Aldrich, 1.0 M in hexanes), Me_2_Zn (Aldrich, 1.0 M in heptane) and all other reagents were of commercial quality and were used without purification. ^1^H NMR, ^13^C NMR spectra were recorded with a Bruker AVANCE 400 spectrometer fitted with BBFO probe with Z gradient. Chemical shifts are reported in δ relative to an internal standard of residual chloroform (δ 7.27 for ^1^H NMR and 77.16 for ^13^C NMR). IR spectra were recorded with an ATR diamond spectrophotometer. High-resolution mass spectra (HRMS) were obtained on a Finnigan MAT 95.

**General Procedure 1. Reaction of *n*-Bu_2_Zn and Et_2_Zn with β-(propargyloxy)enoates 3a and 3b having pendant haloalkynes in the presence of a trace amount of air** (Table 1): Under argon, to a stirred solution of β-(propargyloxy)enoate (0.2 mmol) in Et_2_O (1 mL) at room temperature was added R_2_Zn (0.6 mmol). The reaction mixture was stirred at room temperature for 24 h. The reaction was hydrolyzed with an aqueous solution of HCl (1 M, 10 mL). The layers were separated, the aqueous one being extracted with Et_2_O (2 × 15 mL). The combined organic layers were washed with brine, dried over MgSO_4_, and concentrated under reduced pressure, and the residue was purified by flash chromatography (pentane/ether).

**General Procedure 2. Reaction of *n*-Bu_2_Zn and Et_2_Zn with β-(propargyloxy)enoate 3a in the presence of added air** (Table 2): Under argon, to a stirred solution of β-(propargyloxy)enoate **3a** (0.2 mmol) in CH_2_Cl_2_ (2 mL) at −30 °C was added R_2_Zn (0.6 mmol). Air (2 mL) was bubbled at once into the solution via a syringe fitted with a CaCl_2_ guard and the reaction mixture was stirred at −30 °C for 1 h. CH_2_Cl_2_ (20 mL) and an aqueous solution of HCl (1 M, 10 mL) were added to quench the reaction. The layers were separated, the aqueous one being extracted with Et_2_O (2 × 15 mL). The combined organic layers were washed with brine, dried over MgSO_4_, and concentrated under reduced pressure to afford the crude product.

**(*Z*)-Methyl 4-(bromomethylene)-3-propyltetrahydrofuran-3-carboxylate ((*Z*)-4aa):** Prepared according to general procedure 1 from enoate **3a** (50 mg, 0.2 mmol) and Et_2_Zn (0.6 mL, 1.0 M in hexanes, 0.6 mmol). Purification by flash chromatography with pentane/ether (95:05) as eluent gave the title compound ((*Z*)-**4aa**) (19 mg, 33%) as a colorless oil. IR (neat): 2958, 1730, 1434, 1219, 1072, 938, 722 cm^−1^; ^1^H NMR (400 MHz, CDCl_3_) δ 6.34 (t, *J* = 2.7 Hz, 1H), 4.42 (d(AB system), *J* = 9.1 Hz, 1H), 4.40 (d br, *J* = 2.9 Hz, 2H), 3.84 (d(AB system), *J* = 9.1 Hz, 1H), 3.74 (s, 3H), 1.90 (m, 1H), 1.68 (m, 1H), 1.24 (m, 2H), 0.91 (t, *J* = 7.3 Hz, 3H); ^13^C NMR (100 MHz, CDCl_3_) δ 172.3, 146.6, 100.4, 75.7, 72.8, 58.5, 52.6, 39.5, 18.8, 14.2; HRMS–ESI (*m*/*z*): [M + Na]^+^ calcd for C_10_H_15_O_3_BrNa: 285.00968; found: 285.00990.

**(*E*)-Methyl 4-(bromomethylene)-3-propyltetrahydrofuran-3-carboxylate ((*E*)-4aa):** Prepared according to general procedure 2 from enoate **3a** (47 mg, 0.2 mmol) and Et_2_Zn (0.6 mL, 1.0 M in hexanes, 0.6 mmol). The title compound ((E)-**4aa**) was isolated pure (47 mg, 89%) as a colorless oil and did not require further purification. IR (neat): 2960, 2873, 1769, 1730, 1433, 1217, 1122, 1074, 968, 938, 742 cm^−1^; ^1^H NMR (400 MHz, CDCl_3_) δ 6.15 (t, *J* = 1.9 Hz, 1H), 4.39 (dd(ABX system), *J* = 12.7, 1.9 Hz, 1H), 4.32 (dd(ABX system), *J* = 12.7, 1.8 Hz, 1H), 4.12 (d(AB system), *J* = 8.9 Hz, 1H), 3.97 (d(AB system), *J* = 8.9 Hz, 1H), 3.74 (s, 3H), 2.13 (td, *J* = 13.5, 4.6 Hz, 1H), 1.90 (dt, *J* = 12.5, 4.4 Hz, 1H), 1.50–1.30 (m, 1H), 1.30–1.25 (m, 1H), 0.95 (t, *J* = 7.3 Hz, 3H); ^13^C NMR (100 MHz, CDCl_3_) δ 173.3, 146.7, 98.5, 78.3, 73.8, 58.3, 52.7, 34.7, 17.9, 14.8; HRMS–ESI (*m*/*z*): [M + Na]^+^ calcd for C_10_H_15_O_3_BrNa: 285.0097; found: 285.0091.

**(*Z*)-Methyl 4-(iodomethylene)-3-pentyltetrahydrofuran-3-carboxylate ((*Z*)-4bb):** Prepared according to general procedure 1 from enoate **3b** (40 mg, 0.14 mmol) and Bu_2_Zn (0.42 mL, ~1 N in heptane, 0.42 mmol). Purification by flash chromatography with pentane/ether (80:20) as eluent gave the title compound ((*Z*)-**4bb**) (28 mg, 58%) as a colorless oil. IR (neat): 2951, 2925, 2856, 1731, 1434, 1230, 1073, 939, 730 cm^−1^. ^1^H NMR (400 MHz, CDCl_3_) δ 6.36 (t, *J* = 2.6 Hz, 1H), 4.49 (d(AB system), *J* = 9.1 Hz, 1H), 4.31 (d br, *J* = 2.6 Hz, 2H), 3.90 (d(AB system), *J* = 9.1 Hz, 1H), 3.74 (s, 3H), 1.90 (m, 1H), 1.66 (m, 1H), 1.40–1.20 (m, 6H), 0.91 (t, *J* = 6.9 Hz, 3H); ^13^C NMR (100 MHz, CDCl_3_) δ 172.3, 152.4, 76.5, 76.2, 71.3, 59.7, 52.7, 37.3, 32.0, 25.1, 22.5, 14.1.

**(*E*)-Methyl 4-(bromomethylene)-3-pentyltetrahydrofuran-3-carboxylate ((*E*)-4ab):** Prepared according to general procedure 2 from enoate **3a** (47 mg, 0.2 mmol) and Bu_2_Zn (0.6 mL, ~1 N in heptane, 0.6 mmol). The title compound ((E)-**4ab**) was isolated pure (55 mg, 93%) as a colorless oil and did not require further purification. IR (neat): 2955, 2927, 2856, 1771, 1733, 1433, 1260, 1230, 1217, 1122, 1074, 936, 910, 731 cm^−1^; ^1^H NMR (400 MHz, CDCl_3_) δ 6.15 (t, *J* = 1.9 Hz, 1H), 4.39 (dd(ABX system), *J* = 12.7, 2.0 Hz, 1H), 4.32 (dd(ABX system), *J* = 12.7, 1.8 Hz, 1H), 4.12 (d(AB system), *J* = 8.9 Hz, 1H), 3.97 (d(AB system), *J* = 8.9 Hz, 1H), 3.74 (s, 3H), 2.15 (td, *J* = 13.7, 4.2 Hz, 1H), 1.91 (dt, *J* = 12.2, 4.4 Hz, 1H), 1.50–1.20 (m, 6H), 0.88 (t, *J* = 7.0 Hz, 3H); ^13^C NMR (100 MHz, CDCl_3_) δ 173.3, 146.7, 98.6, 78.3, 73.9, 58.2, 52.7, 32.5, 32.4, 24.1, 22.7, 14.3; HRMS–ESI (*m*/*z*): [M + Na]^+^ calcd for C_12_H_19_O_3_BrNa: 313.0410; found: 313.0413.

**(*E*)-Methyl 4-(bromomethylene)-3-ethyltetrahydrofuran-3-carboxylate ((*E*)-4ac)** (Table 3, entry 1): Under argon, to a stirred solution of β-(propargyloxy)enoate **3a** (46 mg, 0.2 mmol) in CH_2_Cl_2_ (1 mL) at 0 °C was added Me_2_Zn (1 mL, 1.0 M in heptane, 1.0 mmol). Air (20 mL) was slowly introduced over 1 h into the solution via a syringe pump by using a syringe fitted with a CaCl_2_ guard. The reaction mixture was then stirred at 0 °C for 1 h. CH_2_Cl_2_ (5 mL) was then added, and the reaction was hydrolyzed with an aqueous solution of HCl (1 M, 5 mL). The layers were separated, the aqueous one being extracted with CH_2_Cl_2_ (3 × 10 mL). The combined organic layers were washed with brine, dried over MgSO_4_ and concentrated under reduced pressure. Purification by flash chromatography on silica gel (pentane/ether 80:20) afforded the title compound ((E)-**4ac**) (38 mg, 77%) as a colorless oil. IR (neat): 2953, 1767, 1731, 1638, 1229, 1138, 1034, 936, 786 cm^−1^; ^1^H NMR (400 MHz, CDCl_3_) δ 6.17 (t, *J* = 1.9 Hz, 1H), 4.41 (dd(ABX system), *J* = 12.7, 1.9 Hz, 1H), 4.33 (dd(ABX system), *J* = 12.7, 1.9 Hz, 1H), 4.13 (d(AB system), *J* = 8.9 Hz, 1H), 3.98 (d(AB system), *J* = 8.9 Hz, 1H), 3.75 (s, 3H), 2.21 (m, 1H), 2.00 (m, 1H), 0.96 (t, *J* = 7.5 Hz, 3H); ^13^C NMR (100 MHz, CDCl_3_) δ 173.2, 146.4, 98.5, 77.9, 73.8, 58.5, 52.6, 25.2, 8.9; HRMS–ESI (*m*/*z*): [M + Na]^+^ calcd for C_9_H_13_O_3_BrNa: 270.99403; found: 270.99477.

**(*E*)-Methyl 4-(bromomethylene)-3-isobutyltetrahydrofuran-3-carboxylate ((*E*)-4ad)** (Table 3, entry 4): Under argon, to a stirred solution of β-(propargyloxy)enoate **3a** (47 mg, 0.2 mmol) and iPrI (0.2 mL, 2.0 mmol) in CH_2_Cl_2_ (1 mL) at 0 °C was added Me_2_Zn (0.6 mL, 1.0 M in heptane, 0.6 mmol). Air (20 mL) was slowly introduced over 1 h into the solution via a syringe pump by using a syringe fitted with a CaCl_2_ guard. The reaction mixture was then stirred at 0 °C for 1 h. CH_2_Cl_2_ (5 mL) was then added and the reaction was hydrolyzed with an aqueous solution of HCl (1 M, 5 mL). The layers were separated, the aqueous one being extracted with CH_2_Cl_2_ (3 × 10 mL). The combined organic layers were washed with brine, dried over MgSO_4_ and concentrated under reduced pressure. Purification by flash chromatography on silica gel (pentane/ether 50:50) afforded the title compound ((*E*)-**4ad**) (36 mg, 65%) as a colorless oil. IR (neat): 2953, 2870, 1732, 1640, 1230, 1126, 1073, 937, 695 cm^−1^; ^1^H NMR (400 MHz, CDCl_3_) δ 6.15 (t, *J* = 1.9 Hz, 1H), 4.40 (d, *J* = 1.9 Hz, 2H), 4.15 (d(AB system), *J* = 8.9 Hz, 1H), 4.02 (d(AB system), *J* = 8.9 Hz, 1H), 3.74 (s, 3H), 2.05 (m, 2H), 1.77 (m, 1H), 0.96 (d, *J* = 8.4 Hz, 3H), 0.95 (d, *J* = 8.4 Hz, 3H); ^13^C NMR (100 MHz, CDCl_3_) δ 173.1, 146.9, 98.6, 78.0, 73.6, 58.1, 52.5, 40.5, 24.9, 24.5, 24.4; HRMS–ESI (*m*/*z*): [M + Na]^+^ calcd for C_11_H_17_O_3_BrNa: 299.02533; found: 299.02606.

**(*Z*)-Methyl 4-(bromoiodomethylene)-3-ethyltetrahydrofuran-3-carboxylate (16)** (Table 3, entry 2): Under argon, to a stirred solution of β-(propargyloxy)enoate **3a** (45 mg, 0.2 mmol) in CH_2_Cl_2_ (1 mL) at 0 °C was added Me_2_Zn (1 mL, 1.0 M in heptane, 1.0 mmol). Air (20 mL) was slowly introduced over 1 h into the solution via a syringe pump by using a syringe fitted with a CaCl_2_ guard. The reaction mixture was then stirred at 0 °C for 1 h. A solution of I_2_ (330 mg, 1.3 mmol) in THF (1 mL) was then added at the same temperature, and the mixture was stirred for 1 h. CH_2_Cl_2_ (10 mL) followed by an aqueous solution of Na_2_S_2_O_3_ (10%) were added. The layers were separated, the aqueous one being extracted with CH_2_Cl_2_ (2 × 10 mL). The combined organic layers were washed with HCl (1 M) (10 mL) and brine (10 mL), dried over MgSO_4_, and concentrated under reduced pressure. Purification by flash chromatography on silica gel (pentane/ether 80:20) afforded the title compound **16** (46 mg, 64%) as a pale yellow oil. IR (neat): 2947, 2878, 1730, 1630, 1432, 1236, 1081, 941 cm^−1^; ^1^H NMR (400 MHz, CDCl_3_) δ 4.32 (d(AB system), *J* = 14.0 Hz, 1H), 4.27 (d(AB system), *J* = 14.0 Hz, 1H), 4.24 (d(AB system), *J* = 8.8 Hz, 1H), 4.15 (d(AB system), *J* = 8.8 Hz, 1H), 3.75 (s, 3H), 2.21 (m, 1H), 1.95 (m, 1H), 0.98 (t, *J* = 7.5 Hz, 3H); ^13^C NMR (100 MHz, CDCl_3_) δ 172.4, 152.4, 80.1, 79.5, 60.9, 52.8, 41.2, 25.5, 9.0; HRMS–ESI (*m*/*z*): [M + Na]^+^ calcd for C_9_H_12_O_3_BrINa: 396.89067; found: 396.89159.

**(*Z*)-Methyl 4-(bromoiodomethylene)-3-isobutyltetrahydrofuran-3-carboxylate (17)** (Table 3, entry 4): The title compound was obtained as a side-product following the above-described procedure for the preparation of (*E*)-**4ad.** It could not be isolated pure after column chromatography, but characteristic NMR spectroscopic data were obtained. ^1^H NMR (400 MHz, CDCl_3_) δ 4.32 (d(AB system), *J* = 14.0 Hz, 1H), 4.29 (d(AB system), *J* = 14.0 Hz, 1H), 4.24 (d(AB system), *J* = 8.9 Hz, 1H), 4.21 (d(AB system), *J* = 8.9 Hz, 1H), 3.75 (s, 3H), 2.10 (dd, *J* = 14.5, 6.8 Hz, 1H), 1.95 (dd, *J* = 14.5, 5.0 Hz, 1H), 1.81 (m, 1H), 0.98 (d, *J* = 6.7 Hz, 3H), 0.95 (d, *J* = 6.6 Hz, 3H); ^13^C NMR (100 MHz, CDCl_3_) δ 172.5, 152.9, 80.3, 79.8, 58.3, 52.7, 41.9, 40.7, 24.7, 24.5, 23.8.
